# Evolution of Melanopsin Photoreceptors: Discovery and Characterization of a New Melanopsin in Nonmammalian Vertebrates

**DOI:** 10.1371/journal.pbio.0040254

**Published:** 2006-07-25

**Authors:** James Bellingham, Shyam S Chaurasia, Zara Melyan, Cuimei Liu, Morven A Cameron, Emma E Tarttelin, P. Michael Iuvone, Mark W Hankins, Gianluca Tosini, Robert J Lucas

**Affiliations:** **1**Faculty of Life Sciences, University of Manchester, Manchester, United Kingdom; **2**Department of Pharmacology, Emory University School of Medicine, Atlanta, Georgia, United States of America; **3**Nuffield Laboratory of Ophthalmology, University of Oxford, Oxford, United Kingdom; **4**Neuroscience Institute, Morehouse School of Medicine, Atlanta, Georgia, United States of America; Johns HopkinsUnited States of America

## Abstract

In mammals, the melanopsin gene *(Opn4)* encodes a sensory photopigment that underpins newly discovered inner retinal photoreceptors. Since its first discovery in Xenopus laevis and subsequent description in humans and mice, melanopsin genes have been described in all vertebrate classes. Until now, all of these sequences have been considered representatives of a single orthologous gene (albeit with duplications in the teleost fish). Here, we describe the discovery and functional characterisation of a new melanopsin gene in fish, bird, and amphibian genomes, demonstrating that, in fact, the vertebrates have evolved two quite separate melanopsins. On the basis of sequence similarity, chromosomal localisation, and phylogeny, we identify our new melanopsins as the true orthologs of the melanopsin gene previously described in mammals and term this grouping *Opn4m*. By contrast, the previously published melanopsin genes in nonmammalian vertebrates represent a separate branch of the melanopsin family which we term *Opn4x*. RT-PCR analysis in chicken, zebrafish, and *Xenopus* identifies expression of both *Opn4m* and *Opn4x* genes in tissues known to be photosensitive (eye, brain, and skin). In the day-14 chicken eye, *Opn4m* mRNA is found in a subset of cells in the outer nuclear, inner nuclear, and ganglion cell layers, the vast majority of which also express *Opn4x*. Importantly, we show that a representative of the new melanopsins (chicken *Opn4m*) encodes a photosensory pigment capable of activating G protein signalling cascades in a light- and retinaldehyde-dependent manner under heterologous expression in Neuro-2a cells. A comprehensive in silico analysis of vertebrate genomes indicates that while most vertebrate species have both *Opn4m* and *Opn4x* genes, the latter is absent from eutherian and, possibly, marsupial mammals, lost in the course of their evolution as a result of chromosomal reorganisation. Thus, our findings show for the first time that nonmammalian vertebrates retain two quite separate melanopsin genes, while mammals have just one. These data raise important questions regarding the functional differences between Opn4x and Opn4m pigments, the associated adaptive advantages for most vertebrate species in retaining both melanopsins, and the implications for mammalian biology of lacking Opn4x.

## Abstract 2

A new melanopsin gene, identified in fish, bird, and amphibian genomes, is the true ortholog of the melanopsin gene previously described in mammals.

## Introduction

Photoreception within the mammalian retina is not restricted to the activity of rod and cone cells but extends to a small number of intrinsically photosensitive retinal ganglion cells (ipRGCs) [ 1, 2]. These inner retinal photoreceptors provide information regarding environmental irradiance for a variety of non–image-forming light responses including circadian entrainment and the pupillary light reflex [ 3– 6]. A growing body of evidence now implicates a member of the opsin grouping of G protein–coupled receptors, melanopsin, as the photopigment used by these ipRGCs to transduce light into a neuronal signal. Thus, ipRGCs are the primary sites of melanopsin expression [ 7– 9], targeted disruption of the melanopsin gene abolishes direct light responses in these cells [ 10], and human and mouse melanopsins can form functional photopigments under heterologous expression [ 11– 13]. Just like the well-characterised rod and cone opsins, mammalian melanopsins appear to absorb light through a retinaldehyde cofactor and drive changes in membrane potential via G protein signalling cascades.

Although the melanopsin system of mammals has received most recent interest, the founding member of this new branch of the opsin gene family was in fact isolated from the photosensitive dermal melanophores of Xenopus laevis [ 14]. Since then, related sequences have been identified not only in human and mouse [ 15] but also in fish [ 16, 17], bird [ 18], and lizard [ 19] genomes.

Until now it has been assumed that the melanopsin-like sequences in all vertebrate classes are orthologs of the original *Xenopus* melanopsin gene. They cluster together in phylogenetic analyses and share specific structural features. However, while comparisons of deduced amino acid sequence for the published melanopsins reveal significant similarity across species (around 55% identity excluding the N- and C-termini) [ 16], they are much less conserved than comparable sequences in rod and cone opsin families [ 20] (e.g., approximately 85% identity between human and *Xenopus* rod opsins). Until now, we have assumed that an explanation for this relative lack of sequence conservation would come from detailed analyses of melanopsin's sensory and/or signalling capabilities in a variety of vertebrate species. Should significant functional differences between species be identified, the sequence differences could be explained in terms of divergent evolution. Alternatively, should the details of melanopsin's activity be retained across species, one would tentatively conclude that some aspect of its structure renders it accommodating of alterations in an unusually high proportion of amino acid residues. However, a simple alternative to both of these explanations that has not been widely considered is that the published melanopsin sequences are not in fact orthologous and that multiple melanopsin genes have evolved in the vertebrate genome.

We addressed this latter possibility by searching for additional melanopsin-like genes within a variety of vertebrate species. We were successful in identifying new melanopsin genes in chicken, teleost, and *Xenopus* genomes. These sequences reveal the unexpected existence of two quite distinct melanopsin genes in diverse nonmammalian vertebrates. One of these genes, termed *Opn4m*, includes all of our newly described sequences and, in fact, represents the true ortholog of human melanopsin. The previously published melanopsin sequences from nonmammals are actually representatives of a different gene *(Opn4x).* Interestingly, this *Opn4x* gene does not appear in the mammalian genome, having apparently been lost during the evolution of this class. An analysis of the newly described *Opn4m* gene in nonmammals confirms that it is expressed in photosensitive tissues and encodes a sensory photopigment. Our findings raise the possibility of unexpected sensory complexity in melanopsin signalling across the vertebrates.

## Results

### Identification of a Second Melanopsin Locus in Chicken

We commenced our search for additional melanopsin sequences by screening the chicken genome database ( http://www.ncbi.nlm.nih.gov/genome/seq/GgaBlast.html). In order to exclude the known chicken melanopsin gene, we initially queried it with the published chicken melanopsin cDNA sequence [ 18] using the BLASTN algorithm and localised this gene to chromosome 4. We next searched with the amino acid sequence of human OPN4 [ 15] using the TBLASTN algorithm. Among the hits from this screen was a Chromosome 6 contig (NW_060392) that possesses at least five regions of greater than 72% sequence identity (range, 72% to 81%) with human OPN4, suggesting that it represents a novel melanopsin-like protein. To further investigate this possibility, we generated a theoretical partial cDNA sequence by mapping the exonic sequence of human *OPN4* to this chicken genomic contig. This sequence shared significant identity with human OPN4 and, to a lesser extent, the original chicken melanopsin (unpublished data).

**Figure 1:**
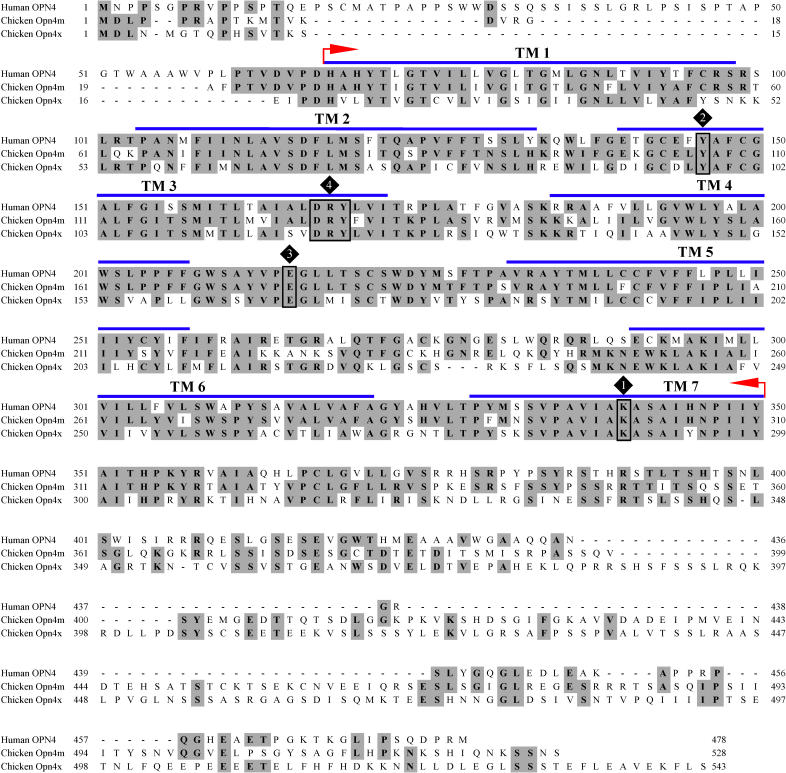
The Deduced Amino Acid Sequence of the New Chicken Melanopsin (Opn4m) Aligned against Human Melanopsin (OPN4) and the Previously Published Chicken Melanopsin Sequence (Opn4x) The “core” region that contains the seven probable transmembrane (TM) domains (blue bars) as predicted by the rod opsin model of Palczewski et al. [ 56] is defined by the red arrows. Numbered diamonds indicate the retinal attachment site, K300 (1); potential Schiff base counterions, Y106 and E175 (2 and 3, respectively); and D127/R128/Y129 tripeptide (4); see text for more details. Shaded residues indicate residue conservation between at least two of the three melanopsins.

In order to verify our predicted sequence, we used it to probe the University of Delaware Chick EST Database ( http://www.chickest.udel.edu) and identified a clone (pgp2n.pk008.h20) from a normalised cDNA library derived from pituitary/hypothalamus/pineal. The sequence of this clone included a 1,587–base pair (bp) open reading frame corresponding to a predicted 528-amino-acid protein (GenBank AY882944). Comparison of this deduced amino acid sequence with that of the published chicken melanopsin ( Figure 1) indicates that it represents a new melanopsin-like locus in this species. Importantly, we were able to verify the nucleotide sequence of this gene by PCR amplification from chicken retinal cDNA.

### The New Chicken Gene *(Opn4m)* Is the True Ortholog of Human Melanopsin

We next set out to compare the predicted amino acid sequence of the novel chicken melanopsin with that of other known melanopsins. It has been shown previously [ 16] that the level of amino acid identity between melanopsins from different vertebrate species is quite low (34% to 60%) when entire sequences are included but significantly higher (54% to 82%) when comparisons are restricted to the “core” region, i.e., that portion predicted to encode the seven transmembrane domains and associated intracellular and extracellular loops but excluding N- and C-termini. Consequently, we compared our new melanopsin gene with known sequences over this central region ( Table 1). Strikingly, the correspondence was always higher for comparisons against the mammalian melanopsins (e.g., approximately 74% identity with human OPN4 and approximately 71% with mouse) than against those of nonmammalian vertebrates (e.g., approximately 58% identity with the previously published melanopsins of chicken and X. laevis).

**Table 1:**
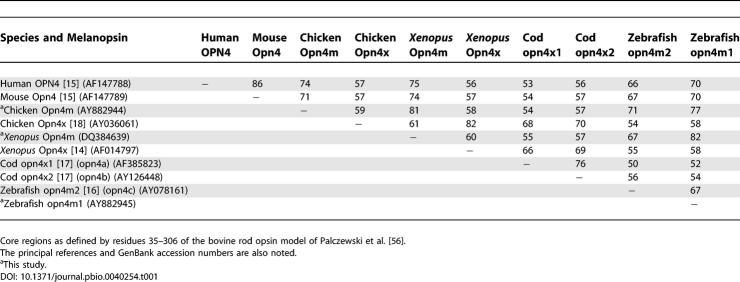
Amino Acid Identity (Percent) across the “Core” Region of Representative Vertebrate Melanopsins

These findings suggest that the new chicken gene may, in fact, be the true ortholog of human *OPN4*. To test this possibility, we set out to compare their chromosomal localisation. We identified human *OPN4* on Chromosome 10q23.2 located in the pter- *KIAA0261-OPN4-LDB3*-qter gene cluster covering approximately 300 kb. An investigation of the chicken genome ( http://www.ensembl.org; Assembly WASHUC 1, March 2004) revealed that the new chicken melanopsin gene is localised to a syntenic region of Chromosome 6 encompassing approximately 250 kb. Gene order together with the phase and locations of introns 1 through 9 (allowing for codon insertion-deletion events) is preserved compared to the human (pter- *ENSGALG00000001907*( *Kiaa0261*)– *Opn4m*- *ENSGALG00000001977*( *Ldb3*)-qter) (see later). On this basis, we conclude that our newly discovered chicken melanopsin gene is in fact the true ortholog of human melanopsin and name it *Opn4m* (denoting “mammal-like”).

For clarity, we suggest that the original chicken melanopsin gene be renamed *Opn4x* (for “ *Xenopus*-like”) on the basis of its greater similarity to the originally published X. laevis melanopsin sequence [ 14] (approximately 82% over the “core” region, compared with approximately 58% for cOpn4m; Table 1).

### Chicken Opn4m Encodes a Sensory Photopigment

The deduced amino acid sequence of cOpn4m retains several features typical of vertebrate opsins in general as well as some that are characteristic of the known melanopsins ( Figure 1). These include seven putative transmembrane α-helices and a DRY tripeptide motif (D127/R128/Y129) at the top of the third α-helix [ 21]; a lysine residue in the seventh α-helix (K300) in a position corresponding to K296 of bovine rod opsin, which allows binding of the chromophore 11- *cis* retinal via a Schiff base linkage [ 22] and is considered diagnostic for the opsin family; and tyrosine (Y106) and glutamate (E175) residues (positions equivalent to E113 and E181 in bovine rod opsin), both of which are retained throughout the melanopsins and have been proposed as potential counterions for the glutamate Schiff base [ 23, 24].

**Figure 2:**
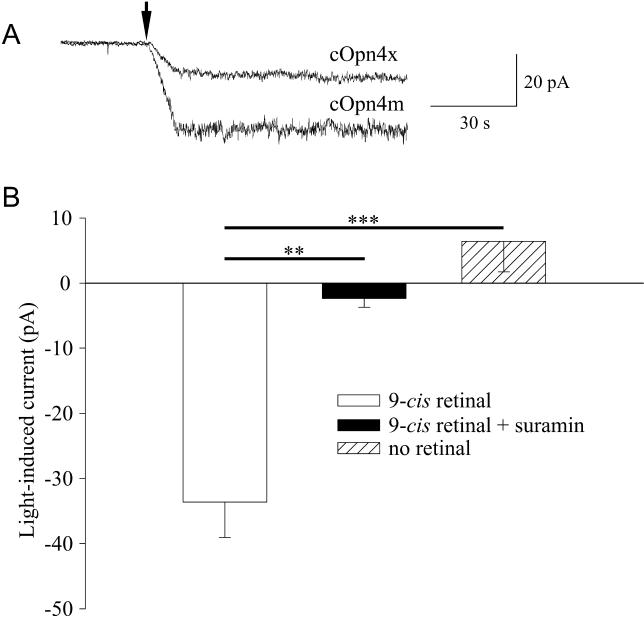
Heterologous Expression of Chicken Opn4m Indicates that It Is a Sensory Photopigment (A) Representative whole-cell patch-clamp recordings from Neuro-2a cells transfected with chicken *Opn4m* or *Opn4x*, in the presence of 9- *cis* retinal exposed to a 420-nm light stimulus at the time indicated by the arrow. (B) Further analysis of the cOpn4m responses revealed that they were abolished by incubation with 100 μM suramin and absent in cells not exposed to retinal (paired *t*-tests, ** *p* < 0.01, *** *p* < 0.0001).

In order to directly determine whether these structural features allow cOpn4m to behave as a sensory pigment, we next adopted the Neuro-2a heterologous expression system recently used to examine human melanopsin [ 11]. Neuro-2a cells were transiently transfected with an expression vector containing the cOpn4m coding sequence and an AcGFP1 reporter. Whole-cell patch-clamp recordings from fluorescent cells revealed light-dependent inward currents in the presence but not the absence of 9- *cis* retinal chromophore ( Figure 2). These currents were effectively blocked by bath application of 100 μM suramin ( *n* = 6), which is effective against the association of G protein α and βγ subunits [ 25]. The importance of G protein signalling for cOpn4m function was confirmed by the observation that inclusion of GTPγS (1 mM) in the patch pipette generated an inward current that precluded subsequent responses to light ( *n* = 3). In all of these aspects, the light-evoked currents observed in the cells transfected with *cOpn4m* are consistent with those obtained with human melanopsin in this expression system and suggest that cOpn4m is a G protein–coupled retinaldehyde-dependent sensory photopigment. We also recorded light-dependent currents from cells expressing cOpn4x, although these were less reproducible and of lower amplitude than those obtained with cOpn4m.

### *Opn4m* in Zebrafish and *Xenopus*

The presence of a second melanopsin gene in the chicken genome, orthologous to human melanopsin, led us to examine the genomes of other nonmammalian vertebrates for evidence of an *Opn4m* gene.

At the time that we commenced this study, members of the Teleostii were the only vertebrates known to have more than one melanopsin gene. The three *opn4* genes in the teleost genome ( *opn4a*, *opn4b,* and *opn4c*) [ 16, 17, 26] were thought to be further examples of the duplication events common for teleost rod and cone opsins [ 27– 32]. When we screened the zebrafish genome, *Danio rerio* (http://www.ncbi.nlm.nih.gov/BLAST), with *cOpn4m*, we identified a potential fourth teleost melanopsin locus on genomic clone CH211-199I23. PCR primers designed against this sequence (opn4dF1 5′- GCCCATTACACTATTGGTGCCG-3′ and opn4dR3 5′- CGGTCCAGCCAGATTCACTATC-3′) were used to amplify a 1,072-bp fragment from zebrafish retinal cDNA. This partial coding sequence is predicted to encode the entire “core” region and a portion of the C-terminal tail of a melanopsin-like gene. Including just the “core” region, it shows 70% amino acid identity with human OPN4 but only 58% with X. laevis Opn4x ( Table 1). As this new locus also has an exon/intron structure conserved with human *OPN4* and *cOpn4m* (unpublished data), we suggest that it is a strict *Opn4m* ortholog.

We finally set out to determine whether Opn4m was present in X. laevis, the species from which the first melanopsin sequence was reported [ 14]. A TBLASTN screen of the Xenopus tropicalis genome ( http://www.ensembl.org/Xenopus_tropicalis/index.html) revealed a contig scaffold_660 that contained regions of identity in excess of 84% to the *cOpn4m* sequence. Fitting the *cOpn4m* and human *OPN4* exon sequence to this contig enabled us to generate a partial predicted *X. tropicalis Opn4m* coding sequence. We used this hypothetical sequence to design primers to be applied to X. laevis eye cDNA under nonstringent conditions. Using primers XentropOpn4mF6 (5′- GACGGTTGATGTTCCAGACC-3′) and XentropOpn4R3 (5′- AGTGGCTGGTAACAGTGGAACG-3′), we were able to amplify and clone a X. laevis–specific 962-bp fragment with a predicted translation of 320 amino acids (GenBank DQ384639). At the amino acid level, the “core” region sequence shows only 60% identity with the original X. laevis melanopsin but 81% to cOpn4m. Hence, it would appear that X. laevis also possesses both *Opn4x* and *Opn4m* genes.

### *Opn4m* versus *Opn4x*

**Figure 3:**
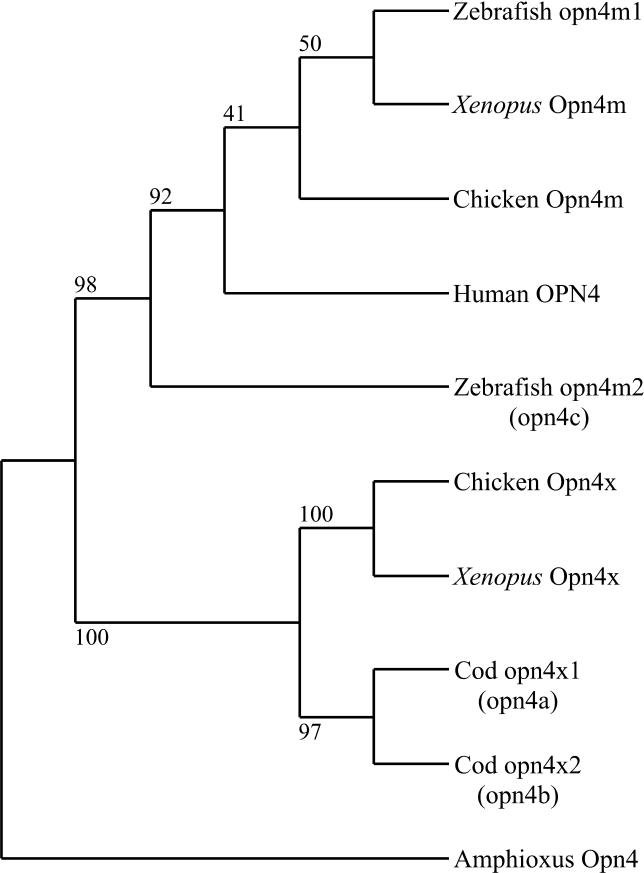
A Revised Phylogeny and Nomenclature of the Vertebrate Melanopsin Family A maximum parsimony phylogenetic tree (derived from amino acid sequences 310 sites, 151 informative, rooted with Amphioxus Opn4) showing the relationship between the novel nonmammalian Opn4m melanopsins and previously published Opn4x melanopsin sequences. Branch confidence levels (% based on 500 bootstrap replicates) reveal an evolutionarily ancient split into the Opn4m and Opn4x branches. The teleost melanopsin nomenclature previously suggested by Drivenes et al. [ 17] is shown in parenthesis; see text for more details.

A phylogenetic analysis of melanopsins from all vertebrate classes supports our contention that this phylum evolved quite separate *Opn4m* and *Opn4*x genes ( Figure 3). Rooting with melanopsin from the protochordate amphioxus [ 33] reveals distinct *Opn4m* and *Opn4x* lineages. This analysis also suggests a reappraisal of the three previously published teleost melanopsins. Two of the teleost melanopsins ( *opn4a* and *opn4b*) fall within the *Opn4x* branch, and we suggest renaming them *opn4x1* and *opn4x2* to maintain consistent nomenclature throughout the vertebrates. The third, *opn4c*, groups with the *Opn4m* melanopsins. As *opn4c* lacks introns [ 34], we tentatively conclude that it has arisen from our newly discovered zebrafish *opn4m* gene, perhaps by retrotransposition [ 27]. On this basis, we suggest naming our new gene *opn4m1* and the original *opn4*c gene *opn4m2*. The presence of *opn4m1* in the zebrafish genome confirms the previous suggestion that teleost *opn4m2 (opn4c)* is not a strict ortholog of the mammalian melanopsins [ 26].

A global comparison of deduced amino acid sequences over the “core” region (excluding N- and C- termini) of melanopsins from representative vertebrate species further confirms the distinction between *Opn4x* and *Opn4m* genes ( Table 1). Within each of the Opn4m and Opn4x groups, identity is relatively high (70% or greater and greater than 66%, respectively), while between the two groups, identity is lower at approximately 55%.

### Mammals Have “Lost” a Melanopsin Gene

**Figure 4:**
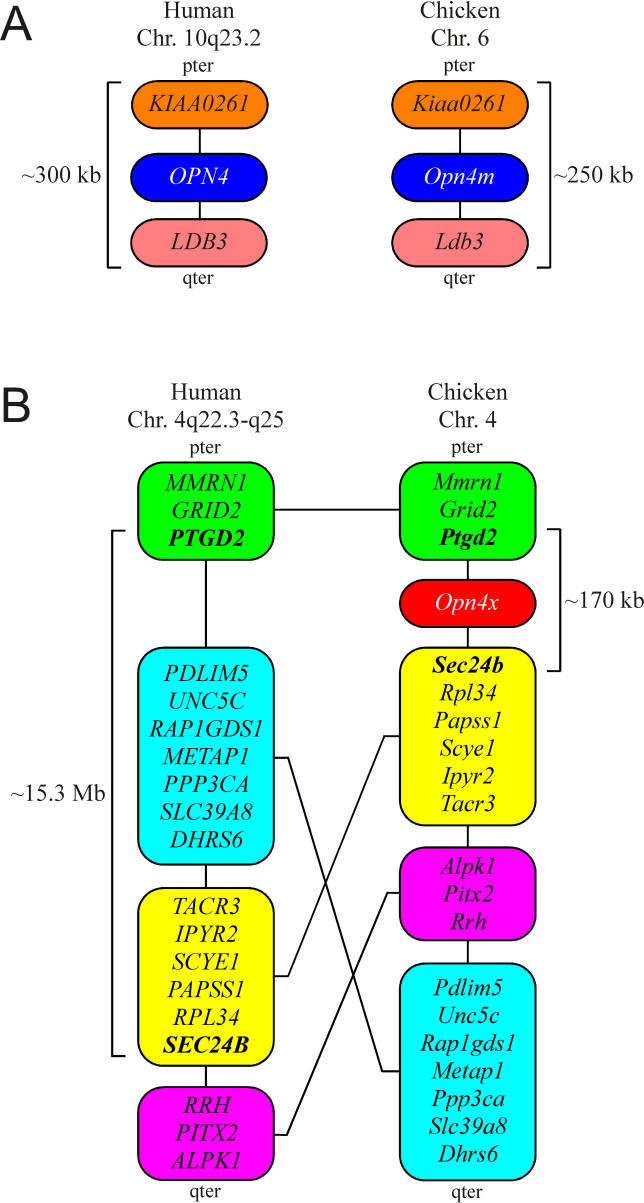
Schematic Diagrams Detailing the Chromosomal Regions Surrounding the Human and Chicken Melanopsin Loci (A) Comparison of syntenic regions encompassing the *OPN4* locus on human chromosome 10 with that of the *Opn4m* locus on chicken Chromosome 4. Gene order has been conserved. (B) Comparison of representative loci between the *PTGD2* and *SEC24B* genes on human Chromosome 4q22.3-q25 (approximately 1 Mb apart) and the orthologous loci on chicken Chromosome 4. Note the intrachromosomal rearrangements and lack of an *OPN4X* locus in humans.

The presence of two distinct melanopsin genes in three separate vertebrate classes ( Figure 3) suggests that this divergence occurred early in vertebrate evolution, prior to the emergence of terrestrial vertebrates. In contrast to the syntenic conservation exhibited by the chicken *Opn4m* and human *OPN4* loci (see earlier and Figure 4A), extensive searching of genome databases for both eutherian and marsupial *(Monodelphis domestica)* mammals ( http://www.ensembl.org) failed to identify an *Opn4x* gene in any member of this class. To further investigate this apparent lack of a mammalian *Opn4x* ortholog, we set out to determine a detailed genomic localisation for *Opn4x* in chicken ( http://www.ensembl.org; Assembly WASHUC 1, March 2004) to compare with syntenic regions of the human genome ( http://www.ensembl.org). *cOpn4x* was localised to chicken Chromosome 4 in a region of approximately 170 kb that encompasses the following immediately adjacent gene cluster pter- *Ptgd2-cOpn4x-ENSGALG00000010414*( *Sec24b*)-qter. The human orthologs of *Ptgd2* and *Sec24b* localise to human Chromosome 4q22.3 ( *PTGD2*) and 4q25 ( *SEC24B*) and are separated by approximately 15.1 Mb. Analysis of this 15-Mb interval between *PTGD2* and *SEC24B* reveals several regions that are syntenic with portions of chicken Chromosome 4 adjacent to the *Opn4x* locus (see Figure 4B). However, their order has been altered, suggesting significant intrachromosomal rearrangements in this region. Importantly, there is no “ *OPN4X*” locus in this portion of human Chromosome 4 (or anywhere else in the genome), suggesting that the ancestral mammalian *Opn4x* locus was deleted during this chromosomal reorganisation.

### Expression of *Opn4m* in Nonmammals

In mammals, melanopsin expression is restricted to a subset of intrinsically photoreceptive retinal ganglion cells [ 7, 15] (although see [ 35, 36]). This very discrete expression pattern contrasts with the wider range of tissues (retina, retinal pigment epithelium, iris, pineal, brain, and skin) reported to express melanopsin in nonmammals [ 14, 17, 18]. As the published expression data for nonmammals primarily relate to the *Opn4x* gene, and those for mammals, by necessity, *Opn4m*, we wondered whether this difference might explain the discrepancy in tissue expression. To address this, we set out to determine whether *Opn4m* shows the same restricted expression in nonmammals as it does in mammals. RT-PCR using gene-specific primers revealed that, in fact, the tissue expression profile of the two melanopsin genes was broadly similar ( Table 2). In all species examined (chicken, zebrafish, and X. laevis), *Opn4m* cDNA was detected in the eye and brain. In embryonic chicken and adult *X. laevis,* it was also found in the skin, whereas in the zebrafish, *opn4m1* was not.

**Table 2:**
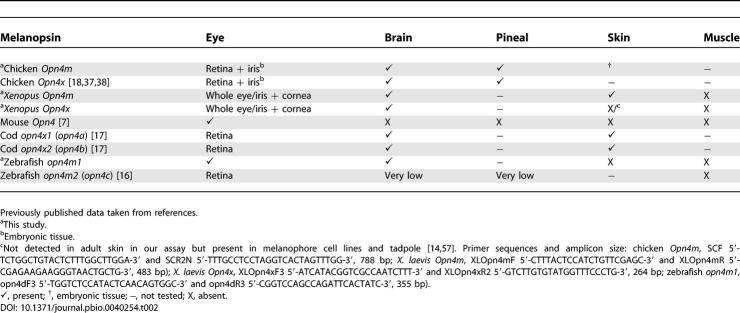
Comparison of the Tissue Expression Pattern of *Opn4m* and *Opn4x* in Various Vertebrate Species as Detected by RT-PCR in This Study or from Previously Published Data

**Figure 5:**
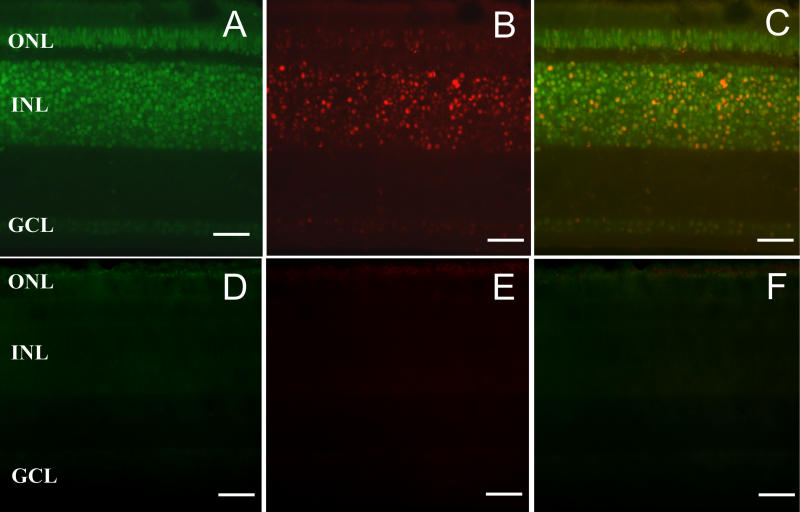
Localisation of *Opn4m* and *Opn4x* Expression in the Chicken Retina Dual in situ hybridisation histochemistry using probes for both *cOpn4x* (fluorescein) and *cOpn4m* (digoxigenin) reveals extensive *cOpn4x* expression [green; (A)], a more restricted pattern for *cOpn4m* [red; (B)], and widespread colocalisation of mRNA for the two genes [yellow; (C), merged image] in sections from 2-wk-old chicken retina. Control sense probes showed no nonspecific labelling [(D), cOpn4x; panel E, cOpn4m; (F), merged image]. ONL = outer nuclear; INL = inner nuclear layer; GCL = ganglion cell layer. Scale bar = 50 μm.

For a more detailed analysis we turned to the chicken retina, in which an especially widespread expression of melanopsin has previously been reported [ 18, 37, 38]. Dual-label fluorescent in situ hybridisation was performed on tissues collected from 2-wk-old animals. Consistent with the RT-PCR data, labelling of the retina occurred with antisense probes (but not sense probes) for both *cOpn4m* and *cOpn4x*. The expression pattern of *cOpn4x* in the chicken retina has been previously documented [ 18, 37, 38], and our findings are consistent with these reports of expression in the ganglion cell layer (GCL), inner nuclear layer (INL), and outer nuclear layer (ONL) ( Figure 5 A). c *Opn4m* mRNA was also detected in all three nuclear layers, with the strongest signal in the inner nuclear layer However, in all three layers, the number of cells positive for c *Opn4m* was much smaller than that positive for *cOpn4x*. A merged image of *cOpn4x* and *cOpn4m* expression ( Figure 5C) reveals significant colocalisation of the two melanopsins with the majority of cells that express *cOpn4m* also expressing *cOpn4x*.

## Discussion

Over the last few years the melanopsin gene has been the subject of intense interest in the fields of vision and circadian biology as evidence has accumulated that it encodes the photopigment responsible for inner retinal photoreception in mammals [ 2, 4, 5, 7– 12, 39]. Nonetheless, the study of melanopsin remains in its infancy compared to that of the well-known rod and cone opsin families. Among the critical issues to resolve are the details of its sensory capabilities and their structural determinants. Although detailed analyses of melanopsin genes from single species will play a critical role in answering these questions (e.g. *,* the native G protein signalling cascade for melanopsin remains unknown in any mammal), experience from the study of rod and cone opsins predicts that comparing melanopsins from diverse vertebrate species will be equally important. Our finding that nonmammalian vertebrates have two quite separate melanopsin genes provides an important change in the conceptual basis for such approaches.

A comparison of previously published melanopsin sequences had revealed relatively low levels of amino acid sequence conservation across species for this protein compared with that reported for other opsin photopigments [ 16]. This had led to speculation either that there might be significant differences in the functional characteristics of melanopsin across species [ 16] or, alternatively, that the nature of structure-function relationships for melanopsin was at some level fundamentally different from those of rod and cone opsins [ 16, 20, 40]. However, our discovery of separate *Opn4m* and *Opn4x* genes provides a simpler explanation of this observation. We observe that amino acid identity over the protein's “core” domain is greater than 66% within the members of each orthologous group even if taken from distantly related species. This is still somewhat lower than that observed within rod and cone opsin families [ 20] (e.g., the long-wave sensitive cone opsins in humans and X. laevis share approximately 81% identity) but significantly higher than previous estimates of melanopsin identity (approximately 50% to 55%) in which both *Opn4m* and *Opn4x* genes were included. Thus, much of the apparent interspecies variation in melanopsin sequence disappears when comparisons are undertaken within *Opn4m* and *Opn4x* genes, revealing differences between these two genes as the major source of sequence divergence among melanopsins.

As sequence differences are greatest between Opn4m and Opn4x proteins, our findings highlight the need for detailed comparisons of functional characteristics between these two melanopsins. The discovery of both genes in diverse vertebrate genomes (fish, bird, and amphibian) identifies their divergence as an ancient event probably occurring prior to the emergence of the Tetrapoda in the Late Devonian some 360 million years ago [ 41, 42]. It also implies a strong selective pressure to maintain both melanopsins over evolutionary time and phylogenetic distance. As this selection should be based on functional differences between the two proteins, our findings suggest an unexpected sensory complexity in melanopsin photoreception. Furthermore, they raise the possibility that a detailed functional characterization of Opn4m and Opn4x proteins to relate to the comparative sequence analysis will provide insights to structure-function relationships in the melanopsins at least as great as those achieved using similar approaches for the rod and cone opsins [ 43, 44].

Our analysis of the *Opn4m* gene in chicken supports the conclusion that it is closely related to the mammalian melanopsins not only in phylogenetic origin but also in function. Under heterologous expression in Neuro-2a cells, cOpn4m behaves similarly to human melanopsin [ 11], activating a native G protein signalling cascade in a light-dependent manner when provided with appropriate isoforms of retinal. The implication of this finding, that cOpn4m forms a sensory photopigment in association with retinal, is supported by its expression in photosensitive tissues.

The function of the Opn4x proteins remains less certain. The restricted expression of this gene in photosensitive tissues and a published report that its overexpression increases light responses in *Xenopus* melanophores [ 45] indicate that it is intimately associated with photoreceptive events. However, our attempt to examine the activity of chicken Opn4x under heterologous expression is the first direct functional examination of this protein from any species. We did observe small light-dependent currents in Neuro-2a cells transfected with *cOpn4x,* suggesting that it, too, encodes a sensory photopigment. However, these were small and less reproducible than those obtained with cOpn4m, and more in-depth analysis of this protein (including, perhaps, expression in other cell types) will be required to confirm this possibility. Assuming that this proves positive and that both cOpn4m and cOpn4x are sensory photopigments, functional differences between the two proteins could arise in their intrinsic sensory capabilities (e.g., spectral sensitivity profile) and/or signalling cascade. At present, direct evidence for either effect is limited, although it is noteworthy that divergent light signalling pathways have been described in the archetypal nonmammalian model for melanopsin studies, the *Xenopus* melanophore [ 46], which could reflect different roles for Opn4x and Opn4m in this system.

We detected *cOpn4m* mRNA by in situ hybridisation in a subset of cells in all three nuclear layers of the 2-wk-old chicken retina. While acknowledging the possibilities that cOpn4m protein may show a more restricted distribution and/or that this expression may reflect a discrete phase in retinal development [ 38], this nevertheless represents the widest distribution to be described for the mRNA of any confirmed photopigment within the vertebrate retina. Its function or functions in these cells remain unknown. It would be surprising if cOpn4m regulated membrane excitability directly in all members of such a diverse population. However, its activities may be cell-type specific and extend to transducing light information for entraining local circadian clocks and/or regulating the synthesis/release of neuromodulators [ 47].

Given the quality of data available for several mammalian genomes, we can be relatively confident in our conclusion that Opn4x is lacking in diverse eutherian mammals. This observation, combined with our failure to find *Opn4x* in a marsupial genome *(Monodelphis domestica),* suggests that this gene was lost relatively early in mammalian evolution, perhaps even predating the separation of metatheria and eutheria in the Jurassic. The loss of *Opn4x* has parallels with two other events related to photoreception which appear to have characterised the early evolution of this class: a simplified colour vision capacity associated with the loss of two cone opsin genes [ 48] and the abolition of extraretinal photoreceptors [ 49]. Together they suggest that early mammals experienced a general reduction in photosensory capability. The precise changes in selective pressure which brought this about will always be a matter of speculation, but an attractive explanation is that it coincided with a nocturnal phase of mammalian evolution [ 50]. In this context, it will be interesting to assess the presence/activity of *Opn4x* and *Opn4m* genes in nonmammalian species adapted to light-limited environments such as snakes, geckos, and deep-sea fish.

Melanopsin was initially discovered in *Xenopus* [ 14] and, partly because of the difficulties associated with its extremely restricted expression pattern in mammals, experiments in nonmammalian species continue to provide an attractive approach to studying the function and physiology of this protein [ 45, 46, 51]. Until now, such studies have been undertaken in the assumption that all vertebrates have a single, orthologous melanopsin gene. The discovery of two quite separate melanopsins requires a reassessment of these strategies. It should certainly precipitate a reinterpretation of published work to address the possible contribution of Opn4m to the results obtained. However, our findings also provide a new impetus for comparative analyses of this sort. In particular, it will be important both to explore the potential for unexpected complexity in melanopsin activity in nonmammals and to determine the consequences of lacking Opn4x on the sensory capabilities of extant mammalian species, including our own.

## Materials and Methods

### In silico database searches.

We have used the protein and nucleotide sequences of previously characterised melanopsins to screen online genome databases ( http://www.ncbi.nlm.nih.gov/BLAST and http://www.ensembl.org/index.html) using the BLAST algorithms [ 52]. Searches were carried out using default values with the low complexity filter off. Subsequent sequence manipulations utilised the online BLAST 2 Sequences [ 53] ( http://www.ncbi.nlm.nih.gov/blast/bl2seq/bl2.html) and MacVector 8.1 (Accelrys).

### Sequence and phylogenetic analysis.

Assembly of predicted sequences, sequence analysis, and identity comparisons were undertaken using MacVector 8.1 (Accelrys). For phylogenetic purposes, amino acid sequences were aligned with ClustalX 1.83 ([ 54]; ftp://ftp-igbmc.u-strasbg.fr/pub/ClustalX) and maximum parsimony trees were constructed using Phylo_win ([ 55]; http://pbil.univ-lyon1.fr/software/phylowin.html).

### RNA extraction, cDNA synthesis, and PCR.

Tissue was collected from adult zebrafish *(Danio rerio)* and X. laevis and 14-d-old chickens (male *Gallus gallus domesticus*; obtained from Hyline International, Covington, Georgia, United States, and housed at Emory University) for all analyses, except for the chicken iris and skin RT-PCR experiments, which utilised 16-d-old chicken embryos. Total RNA was isolated using commercial kits (TRI Reagent, Sigma-Aldrich, St. Louis, Missouri, United States; RNeasy, Qiagen, Valencia, California, United States) according to the manufacturer's instructions and treated with RNase-free DNase I (Promega, Madison, Wisconsin, United States) prior to cDNA synthesis using the SuperScript system (Invitrogen, Carlsbad, California, United States) and employing either random hexamers or oligo-dT primers. All subsequent PCRs employed primer pairs designed to amplify across at least one intron to exclude genomic DNA contamination. Positive controls were used to confirm cDNA quality (zebrafish β-actin, ZFBactinF 5′- ATGGAGAAGATCTGGCATCA-3′ and ZFBactinR 5′- ACGGAAACGCTCATTGCCGAT-3′, 528 bp; X. laevis glyceraldehyde-3-phosphate dehydrogenase, *Gapdh*, XgapF 5′- AGGGAACCGTTAAGGCTGAG-3′ and XgapR 5′- GACTGTTGTCATGAGTCC-3′, 365 bp; chicken *Gapdh*, CHGAPDHF 5′- ACCACTGTCCATGCCATCAC-3′ and CHGAPDHR 5′- TCCACAACACGGTTGCTGTA-3′; 452 bp). Amplicons were analysed by agarose gel electrophoresis and, where necessary purified and sequenced to confirm their identity.

### In situ hybridisation.

To minimise the possibility of cross-hybridisation of in situ probes, PCR primers were designed to amplify a 322-bp fragment encompassing the 272-bp 5′ UTR and first 50 bp of coding sequence of the *cOpn4m* gene (Chick Opn4M 5′ UTR F1: 5′- GCTCTAGAGCAAGCTGAAGCTCTGGTGAG-3′ and Chick Opn4M Exon2 R1: 5′- CCCTCGAGCGAACATCTTTCACTGTCATC-3′), and a 338-bp fragment of the *cOpn4x* 3'UTR (Chick Opn4X 3′ UTR F1: 5′- GCTCTAGACCAAATAACCGAAGCCTTGTAGG-3′ and Chick Opn4X 3′ UTR R1: 5′- CCCTCGAGTTACCACCACCTCCAGAAGAGAGC-3′). BLAST searches of the chicken genome with these two UTR sequences indicated that they are specific for their respective genes. Primers were tagged with XbaI and XhoI sites (underlined) to allow directional cloning of the PCR products into pBluescript II SK+ (Stratagene, La Jolla, California, United States). The resulting plasmids were sequenced to ensure probe fidelity. Antisense and sense cRNA probes were generated by in vitro transcription incorporating fluorescein-12-UTP (PerkinElmer Life Sciences, Boston, Massachusetts, United States) for the *cOpn4x* probes and digoxigenin-11-UTP (Roche, Basel, Switzerland) for the *cOpn4m* probes. Retinal sections (14 to 16 μM) from 2-wk-old chickens (HyLine International, Covington, Georgia, United States) were immersed in prehybridisation buffer containing 50% formamide, 5× Denhardt's solution, and 5× SSC for 2 h at 22 °C. Then the sections were hybridised with 75 μl of hybridisation buffer including the *cOpn4m* and *cOpn4x* probes, cover-slipped, and incubated overnight in a humidified chamber at 61 °C. Optimal labelling was obtained using a probe concentration of 1:100. Slides were then washed in 5× SSC at 68 °C for 30 min// and then in 0.2× SSC at 68 °C for 1 h prior to incubation in 20 mg/ml RNase A at 37 °C for 30 min. Further posthybridisation stringency washes in 0.2× SSC for 1 h at 68 °C (once) and 0.2× SSC for 30 min at 22 °C (twice) were followed by a 30-min incubation in blocking buffer at 22 °C and then incubation with antidigoxigenin-POD (Roche) at 1:100 overnight at 4 °C. The slides were then washed for 5 min in PBS buffer at 22 °C (three times) and then incubated in TSA Cyanine 3 Amplification Reagent (PerkinElmer) working solution for 7 min at 22 °C before being washed again (three times in PBS for 5 min at 22 °C) and then mounted for fluorescence microscopy using Vectashield (Vector Laboratories, Burlingame, California, United States).

### In vitro expression of chicken *Opn4m* and *Opn4x*

Full-length coding sequences for chicken *Opn4m* and *Opn4x* were amplified from retinal cDNA using Phusion High-Fidelity DNA Polymerase (Finnzymes/New England Biolabs, Beverly, Massachusetts, United States). PCR products were cloned into pCR4Blunt-TOPO (Invitrogen) prior to sequence verification and eventual cloning into the expression vector pIRES2-AcGFP1 (BD Biosciences, San Diego, California, United States). Plasmid DNA for pIRES2-AcGFP1-cOpn4m and pIRES2-AcGFP1-cOpn4x to be used in transfections was prepared with a HiSpeed Plasmid Maxi Kit (Qiagen).

Mouse Neuro-2a cells were maintained, transfected, and differentiated as previously described [ 11]. Successfully transfected cells were identified under appropriate AcGFP1 fluorescence using an Olympus BX51WI microscope. Then 20 μM 9- *cis* retinal (Sigma) was added to the perfusion solution, and cells were kept in the dark for at least 1 h before recording. Whole-cell patch-clamp recordings were made with pipettes containing 140 mM KCl, 10 mM NaCl, 1 mM MgCl _2_, 10 mM HEPES, and 10 mM EGTA. Osmolarity was adjusted to 285 ± 5 mosmol/L and pH to 7.3 to 7.4 with KOH. Open pipette resistance was 2 to 5 MΩ, and access resistance during recordings was less than 20 MΩ. Currents were recorded (Axopatch 200B, Axon Instruments, Union City, California, United States) in neurons voltage clamped at holding potentials of −50 mV. The records were filtered at 1 kHz and sampled at 20 kHz. Suramin (Sigma) (100 μM) was applied in perfusion solution for 15 to 20 min prior to light stimulation. Light stimuli were generated using a Cairn Optoscan Xenon arc source comprising a slit monochromator. All stimuli were 10 s in duration with a 20-nm half-bandwidth. Irradiance was measured using an optical power meter (Macam Photometrics) and converted to photon flux. The magnitude of the responses was defined by the peak sustained current measured using Clampfit (Axon Instruments).

## Supporting Information

### Accession Numbers

The GenBank ( http://www.ncbi.nlm.nih.gov/Genbank) accession numbers used in this paper are human OPN4 (AF147788), mouse Opn2 (AF147789), genomic clone CH211-199I23 (BX897719), chicken Opn4m (528-amino-acid protein) (AY882944), Amphioxus Opn4 (AB205400), chicken Opn4x (chicken melanopsin cDNA sequence) (AY036061), cod opn4x1 (AF385823), cod opn4x2 (AY126448), *Xenopus* Opn4m (320 amino acids) (DQ384639), *Xenopus* Opn4x ( X. laevis melanopsin) (AF014797), zebrafish opn4m1 (zebrafish retinal cDNA) (AY882945), and zebrafish opn4m2 (AY078161).

## Abbreviations

bp: base pairs
ipRGC: intrinsically photosensitive retinal ganglion cell
