# Correction: Evolution of Melanopsin Photoreceptors: Discovery and Characterization of a New Melanopsin in Nonmammalian Vertebrates

**DOI:** 10.1371/journal.pbio.0040320

**Published:** 2006-10-17

**Authors:** James Bellingham, Shyam S Chaurasia, Zara Melyan, Cuimei Liu, Morven A Cameron, Emma E Tarttelin, P. Michael Iuvone, Mark W Hankins, Gianluca Tosini, Robert J Lucas

## Abstract

A new melanopsin gene, identified in fish, bird, and amphibian genomes, is the true ortholog of the melanopsin gene previously described in mammals.

In *PLoS Biology*, volume 4, issue 8: DOI: 10.1371/journal.pbio.0040254

There was an error in the legend for Figure 4. The text describing part A should read “chicken chromosome 6” rather than “chicken chromosome 4.”

